# Longitudinal course of bone health from infancy to mid-adulthood in preterm and term-born individuals

**DOI:** 10.1038/s41598-025-29584-0

**Published:** 2025-11-26

**Authors:** Julie Brecher, Aziz Chaouch, Daniel Konrad, Dominique A. Eichelberger, Beatrice Latal, Oskar G. Jenni, Nina Lenherr-Taube, Flavia M. Wehrle

**Affiliations:** 1 Child Development Center, University Children’s Hospital Zurich, Zurich, Switzerland; 2 Department of Epidemiology and Health Systems, Quantitative Research, Center for Primary Care and Public Health (Unisanté), University of Lausanne, Lausanne, Switzerland; 3 Children’s Research Center, University Children’s Hospital Zurich, Zurich, Switzerland; 4 University of Zurich, Zurich, Switzerland; 5 Division of Pediatric Endocrinology and Diabetology, University Children’s Hospital Zurich, Zurich, Switzerland

**Keywords:** Bone imaging, Outcomes research, Preterm birth

## Abstract

Preterm birth is associated with various health consequences. However, the long-term impact on bone health remains unclear. This study compares bone health from infancy into mid-adulthood between preterm and term-born individuals and explores potential association with fracture status. Data from the Zurich Longitudinal Studies were analyzed, including preterm and term-born individuals (enrolled 1974–1979). Bone health was measured longitudinally using the Bone Health Index (BHI) from hand X-rays analyzed by BoneXpert. Fracture history was obtained through parental interviews and self-reports. Generalized estimating equation models evaluated BHI over age, adjusting for age, sex, preterm and fracture status. 1582 X-rays from 95 term and 120 preterm individuals, across 17 time points (3 months to 47 years of age) were analyzed. Preterm individuals (median gestational age 34.1 weeks, IQR ± 3.1) had significantly lower BHI than term-born peers (*p* = *0.016*), with a deficit persisting until 4.5 years of age. Thereafter no difference was found. No significant association between BHI and fracture status in mid-adulthood was found *(p* = *0.43).* In this cohort of predominantly moderate to late preterm infants, catch-up in bone health was achieved in early childhood and maintained into mid-adulthood. How bone health and fracture status evolve in later life remains unclear and requires a lifespan perspective on preterm birth.

**Supplementary Information:**

The online version contains supplementary material available at 10.1038/s41598-025-29584-0.

## Introduction

Preterm birth (< 37 weeks of gestation) is a major global health challenge, affecting millions of infants annually^1^. It is linked to an increased risk of various chronic conditions, including impaired lung function, cardiovascular disease, metabolic disorders, and neurodevelopmental challenges^2^. Advances in neonatal care have considerably improved the survival rates of preterm infants^3,4^, resulting in a growing population of individuals at risk for long-term health consequences of preterm birth.

Among the various physiological systems affected by preterm birth, the skeletal is of particular importance, as it plays a crucial role in overall health, mobility, and fracture risk throughout life^5,6^. Fetal bone mineralization occurs predominantly in the third trimester, during which approximately 80% of total body mineral content is acquired through placental transfer^7,8^. Following birth, bone undergoes an intensive remodeling process, characterized by a physiological decrease in bone mineral density (BMD) due to increased resorption to maintain calcium homeostasis. This dynamic process supports skeletal growth and adaptation to mechanical forces^9^. Peak bone mass is typically achieved in late adolescence to early adulthood. It is a key determinant of lifelong bone strength, with higher peak values associated with a reduced risk of osteoporosis and fractures later in life^5,10,11^.

Preterm birth disrupts the critical period of fetal bone mineralization mainly occurring during the third trimester. Additional prematurity-related factors compound the risk of impaired bone health and overall skeletal development, including the need for parenteral nutrition, extended periods of immobilization, or the use of medication such as corticosteroids or diuretics^5,7,12–14^. While it is well-known that preterm infants have an increased risk of metabolic bone disease in early life^7,8,12–14^, only a handful of studies have investigated the long-term consequences.

Cross-sectional studies focusing on cohorts with very low birth weight (VLBW; < 1500 g) or very preterm birth (< 32 weeks of gestation) consistently report lower BMD up to mid-adulthood compared to term-born controls^15–23^. In contrast, studies on moderate to late preterm cohorts (32–36 weeks of gestation) found no impact of preterm birth on BMD or peak bone mass in young adulthood^20–22^ . More recently, reduced BMD at 7–8 years of age has been reported in preterm-born individuals, irrespective of gestational age, when compared with term-born peers^24^. So far, longitudinal studies have been limited to early childhood^25,26^. Mihatsch et al. observed persisting differences in BMD between preterm and term-born infants up to three years, indicating that early BMD catch-up did not occur^25^, whereas Zhao et al. demonstrated that preterm infants exhibited an accelerated BMD growth trajectory, suggesting that early BMD catch-up may be possible for moderately preterm infants^23^. These heterogeneous findings highlight the need for a comprehensive longitudinal approach into adulthood to understand the long-term impact of preterm birth on bone health.

Thus, this study aims to characterize the longitudinal evolution of bone health in preterm compared to term-born individuals from infancy to mid-adulthood and explore the link to fracture status. We hypothesize that preterm individuals have lower bone health in infancy relative to their term-born peers but experience catch-up within the first years of life. Whether this catch-up persists into mid-adulthood will be explored. Additionally, we propose that children with reduced bone health are at a higher risk of fractures across the first five decades of life.

## Methods

### Study design and sample

Data was taken from the Zurich Longitudinal Studies (ZLS), initiated in the 1950s (first cohort) and the 1970s (second and third cohort). The ZLS focused on the growth, development and health of children and adolescents. Since 2019, the data has been extended into mid- and older adulthood^27^.

For the current analyses, data assessed within the second cohort (ZLS-2) was considered, as this cohort included preterm and term-born infants. Between 1974 and 1979, 159 preterm (30 died as neonates) and 99 term-born infants were enrolled at birth (deviations from study protocol^27^ are described in^28^). Gestational age was determined based on the last menstrual period for all infants included^28^. Data was collected at multiple time points across infancy, childhood, and adolescence: at 3, 6, 9, 12, 18, and 24 months, and at 3, 4, 5, 6, 7, 8, 9, 10, 14, and 18 years. Assessments were scheduled around the participants’ birthday. For preterm infants, assessments were conducted at the child’s age, corrected for prematurity^27^. The most recent assessment took place in mid-adulthood between 2019 and 2021, with participants ranging in age from 40 to 47 years. One hundred six individuals participated as adults (49% of the surviving 218 participants who could be traced^28^).

The study is approved by the ethical committee of the Canton of Zurich, Switzerland (Basec No. 2018–00,686) and all analyses were performed in accordance with the WMA Declaration of Helsinki. Informed consent was obtained from participants and their legal guardians. Further details on the informed consent procedure since the initiation of the ZLS in 1954 are provided in Wehrle et al.^27^.

### Measures

Each assessment included a comprehensive, standardized test battery and a set of questionnaires, covering various domains such as physical, motor, cognitive, and social health and development. Also an X-ray of the left hand was taken (for further details on the full study protocol, see Wehrle et al.^27^).

#### Bone health

Bone health was assessed from hand X-rays, using the Bone Health Index (BHI) derived from the BoneXpert software. BoneXpert is an automated software that calculates, among other variables, bone age, BHI, and BHI standard deviation score (BHI-SDS). BHI defines bone health as the biomechanical balance of bone mass adapted to the environment by natural selection to ensure that healthy children have an optimal amount of bone^29^. It is quantified using the formula based on the cortical thickness, width and length of three middle metacarpals: $BHI = \pi T (1-T/W) / {(LW)}^{0.33}$*.*

BoneXpert Standalone software version 3.2.3 (algorithm v1.7.3) for children and adolescents and BoneXpert Adult version 2.3 for adults were used. During the analytical process, X-rays are automatically rejected if analysis is not possible, e.g., due to poor hand positioning or image quality^30^. BHI is independent of body height and is applicable to both pediatric and adult populations. BHI-SDS values are adjusted for bone age and sex; however, if the calculated bone age exceeds 15 years in girls or 17 years in boys, chronological age is used instead to determine BHI-SDS^29,31–33^.

#### Fractures

Information on fractures were collected using parental (semi-)structured interviews at every visit until the age of 14 years. At 18 years, the participants themselves were interviewed. In adulthood, participants answered a questionnaire on types and on time-point of injuries sustained in the past. All answers were manually inspected for any fractures that had occurred and checked for multiple entries of the same fracture. Sprains or other inconclusive information (e.g., “cast”) were not interpreted as fractures, whereas bone fissures were. Events leading to several fractures (such as “car accident causing several fractures”) were counted as one fracture, whereas information such as “multiple fractures of fingers and toes during the last 10 years” was counted as two fractures (one for fingers and one for toes). Participants were identified as potential osteoporotic if they had sustained ≥ 2 fractures of long bones before the age of 10 years, ≥ 3 fractures of long bones before the age of 19 years, or if they had a nontraumatic vertebral compression fracture^34^. For further analysis, participants were classified into two groups: those with no fractures until mid-adulthood and those who had sustained at least one fracture. Importantly, childhood fracture history covers events occurring both before and after measurements of childhood BHI. As a consequence, it cannot be treated as a causal predictor of childhood BHI. However, we still explored the association between fracture history and childhood BHI to assess whether BHI dynamics differ between individuals who eventually sustain a fracture and those who do not. Indeed, later-occurring fractures may serve as a proxy for underlying frailty, which may be present from birth. While this approach has no causal interpretation, it remains informative and was therefore included in this descriptive study.

#### Further variables

To describe sample characteristics, further variables were evaluated. Based on Prechtl’s optimality concept^35^, an optimality score adjusted by Largo^36^ was assessed. This score incorporates factors during pregnancy, birth and the neonatal period, such as infections, bronchopulmonary dysplasia, necrotizing enterocolitis, required medications and other relevant medical conditions. The maximum of 90 points indicates optimal outcome. To account for missing information within a participant, in the current analysis, the optimality score was calculated as the percentage of attained points relative to the total possible points for each participant, i.e., considering only the completed items. Childhood socio-economic status (SES) was assessed using paternal occupation and maternal education, measured on a scale from 2 to 10, with higher scores indicating lower SES, following Largo et al^37^.

#### Statistical analysis

Analysis was performed in R version 4.4.0. Demographic information was presented as percentages for categorical variables, while continuous variables were summarized using mean and standard deviation (SD) or median and interquartile range (IQR) as appropriate. Group differences in continuous variables were analyzed using the independent samples t-test and Mann–Whitney U test, and Chi-square tests or Fisher’s exact tests were used for categorical variables. A generalized estimating equation (GEE)^38^ model with an exchangeable correlation structure, using the geeglm function from the geepack package^39^, was used to investigate bone health evolution. GEE models are a powerful tool for analyzing longitudinal data, as they handle unequal numbers of repeated measurements per individual and allow to account for the correlation structure between those repeated measurements without having to explicitly define it^38,40^. In a first model, BHI was used as the outcome variable, with preterm status, chronological age, sex and the interaction between preterm status and chronological age as predictors. Modeling was conducted using BHI raw values rather than BHI-SDS values, as it allows to directly compare the subpopulations without introducing a third external reference sample. The age effect was modeled using natural cubic splines to capture potential nonlinear trends and the optimal number of degrees of freedom for the nonlinear effect of age was selected based on the lowest uncorrected Quasi-likelihood under the Independence model Criterion (QICu) value, with preference given to the model with fewer degrees of freedom in case of ties^41^.

To identify the age ranges in which BHI values differ significantly between preterm and term-born individuals, we conducted a simulation based on the fitted GEE model. For a given gender, predicted BHI values were calculated separately for preterm and term-born individuals over a thin grid of age. The difference in predicted BHI values (preterm – term-born) was then calculated at each age. A 95% pointwise confidence interval for that difference was calculated by drawing model coefficients based on their asymptotic gaussian sampling distribution and repeating the calculation of the difference in BHI values. A total of 10′000 simulations were carried out to build the confidence interval. The resulting figure depicts the predicted difference in BHI values as a function of age. Age ranges in which the confidence interval does not include the zero line can be interpreted as statistically significant differences. Since bone age is the preferred metric for assessing skeletal maturity in childhood above chronological age, i.e., due to its greater sensitivity to pubertal changes^42^, we also repeated the analysis using bone age instead of chronological age within the available age range. In a second GEE model and simulation, fracture status in mid-adulthood (yes/no) was added to explore potential differences of BHI across age between individuals who had never sustained a fracture and those who had sustained at least one fracture in a descriptive manner.

## Results

### Sample characteristics

A total of 1582 radiographs across 17 time points from 215 participants were analyzed. Individuals contributed between one *(n* = *6)* and 15 *(n* = *6)* radiographs *(Mdn* = *6, IQR* = 9; Supplementary Fig. 1). Of 43 individuals, no radiographs were available, either because they had died as neonates *(n* = *30)* or because of parental refusal to take radiographs *(n* = *13).* The final analytic sample comprised 120 preterm and 95 term-born infants (Table 1).

**Table 1:** Sample characteristics.

	n	Term	n	Preterm	*p* value
Sociodemographic Characteristics					
Sex (%female)	95	47.4%	120	40.8%	0.41
Child socio-economic status (Mdn ± IQR)	95	5 ± 2	120	6 ± 3	0.01
Fracture status in mid-adulthood^a^ (%with at least one fracture)	58	50%	68	36.8%	0.19
Neonatal Characteristics					
Birthweight (gram; Mdn ± IQR)	95	3320 ± 460^b^	120	1,995 ± 663^b^	< 0.001
Gestational Age (weeks; Mdn ± IQR)	95	> 37^c^	109	34.1 ± 3.1	–
< 28 weeks, n (%) 28 + 0 – 31 + 6 weeks, n (%) 32 + 0 – 36 + 6 weeks, n (%)			109	0 (0%) 27 (24.8%) 82 (75.2%)	
Small for gestational age (%)^d^	95	0%	109	2.8%	0.33
Optimality Score (Mdn% ± IQR%)	86	95% ± 4%	69	81% ± 10%	< 0.001
Necrotizing Enterocolitis (%)	85	0%	69	1.4%	0.45
Bronchopulmonary Dysplasia (%)	85	0%	69	2.9%	0.20
Cerebral Hemorrhage (%)	85	0%	69	4.3%	0.09
BHI—SDS values (M ± SD)					
3 months	30	0.52 ± 0.61	41	− 0.14 ± 0.74	< 0.001
6 months	32	0.22 ± 0.63	43	− 0.33 ± 0.82	0.002
9 months	29	0.18 ± 0.63	44	− 0.21 ± 0.79	0.03
1 year	34	0.06 ± 0.65	39	− 0.09 ± 0.70	0.32
1.5 years	43	− 0.02 ± 0.74	42	0.06 ± 0.74	0.60
2 years	39	− 0.17 ± 0.86	54	0.13 ± 1.24	0.18
3 years	43	− 0.29 ± 0.98	50	0.08 ± 1.19	0.11
4 years	50	− 0.21 ± 0.99	50	0.08 ± 1.17	0.19
5 years	47	− 0.09 ± 0.99	53	− 0.04 ± 1.15	0.79
6 years	41	− 0.08 ± 1.03	48	0.00 ± 1.19	0.72
7 years	35	0.00 ± 0.97	51	0.30 ± 1.35	0.23
8 years	20	− 0.18 ± 0.75	32	0.27 ± 1.29	0.12
9 years	8	0.61 ± 0.76	5	0.68 ± 1.32	0.91
10 years	67	0.34 ± 1.09	69	0.26 ± 0.94	0.63
14 years	83	0.20 ± 1.05	100	0.33 ± 1.06	0.41
18 years	83	− 0.03 ± 1.08	99	0.10 ± 0.98	0.39
40–47 years	33	− 0.18 ± 0.77	36^e^	− 0.06 ± 1.01	0.58

^a^One preterm-born participant, who sustained four long bone fractures before the age of 19 years, was classified as potential osteoporotic. No BMD from DXA was available for this individual to confirm the diagnosis.

^b^ none of the term-born individuals, and 17/120 (14.2%) of the preterm born individuals fullfilled criteria of very low birth weight (< 1500g).

^c^ For term-born infants, no further information was available.

^d^ Small for gestational age defined as -2 SD according to Ingeborg Brandt: Human Growth. A Comprehensive Treatise. 2 .Ed. Vol. 1 . Eds. F. Falkner and J. M. Tanner, Plenum Press. New York 1986.

^*e*^ One individual was excluded in mid-adulthood due to gender-affirming therapy, which affects bone health and complicates assignment to male or female normative values.

### Bone health from infancy into mid-adulthood in preterm and term-born individuals

**Fig. 1:**
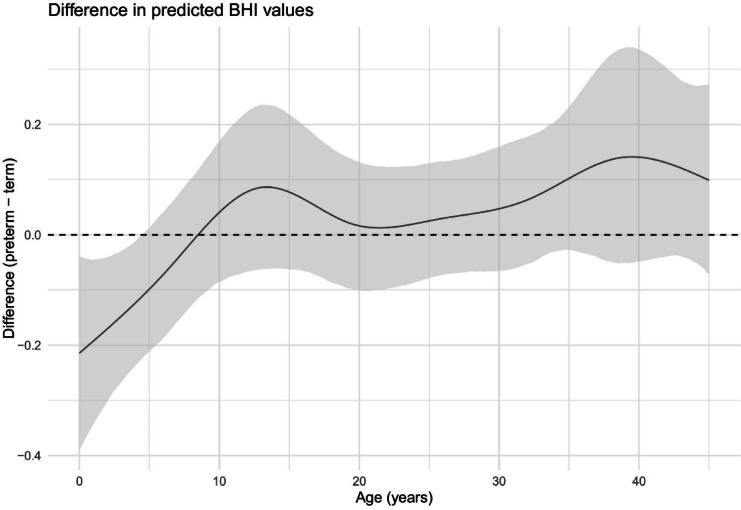
Estimated difference of the predicted BHI values over age based on the GEE model. Solid line: estimated difference. Shaded area: 95% confidence interval. Values above the dashed line indicate higher BHI values for preterm infants. Statistically significant areas have confidence intervals excluding zero.

The best-fitting GEE model used natural cubic splines with 7 degrees of freedom to model the age effect *(QICu* = *294).* The GEE model revealed a significant main effect of preterm birth on BHI, with preterm individuals exhibiting lower BHI compared to term-born individuals *(β* = *-0.215, SE* = *0.089, p* = *0.016).* Sex was not significantly associated with BHI (*β* = *0.033, SE* = *0.045, p* = *0.46).* Age had a significant effect on BHI, following a nonlinear trajectory, with a decrease in BHI during early childhood, followed by an increase in later age segments. Furthermore, significant interactions between preterm status and age splines indicate that preterm and term-born individuals exhibit distinct age-related evolutions in BHI. As illustrated in Fig. 1, the GEE model simulation showed that preterm infants had a significantly lower BHI compared to term-born infants up to the age of 4.5 years. Beyond this age, the difference was no longer significant. Based on the estimated difference, preterm infants, on average, achieve catch-up in BHI by 8.5 years of age. Excluding one individual with potential osteoporotic fractures did not alter the findings (data not shown).

When repeating the analysis across the assessments between infancy and 18 years of age using bone age instead of chronological age, the results remained consistent (Supplementary Fig. 2).

### Association between BHI and fractures

For the following analysis, 891 radiographs from 126 participants with available information on fracture status were included, comprising 68 preterm and 58 term-born individuals.

The best-fitting GEE model, using natural cubic splines with 5 degrees of freedom *(QICu* = *183.9),* showed no significant main effect of fracture status on BHI *(β* = *0.11, SE* = *0.13, p* = *0.41)*. Sex also had no significant effect *(β* = *0.06, SE* = *0.06, p* = *0.30).* However, preterm status significantly affected BHI *(β* = *-0.25, SE* = *0.12, p* = *0.040)*, with preterm infants having lower BHI. Age exhibited a significant non-linear relationship with BHI, with an overall increasing trend as age increased. Significant interaction terms between age and preterm status were observed, indicate that preterm and term-born individuals exhibit distinct age-related evolutions in BHI. In contrast, no significant interaction between fracture status and age was detected. As illustrated in Supplementary Fig. 3, the estimated difference in BHI evolution between individuals with and without fractures remained consistently non-significant across age. Notably, the inclusion of fracture status led to an increase in QICu values *(QICu* = *183.9)* compared to the model without fracture status *(QICu* = *172.9)*, indicating that fracture status did not improve the model fit. Excluding one individual with potential osteoporotic fractures did not affect the results (data not shown).

## Discussion

This study evaluated the longitudinal evolution of bone health in predominantly moderate to late preterm and term-born infants over five decades, from infancy to mid-adulthood, and explored the association between bone health and fracture status.

Our results indicate that bone health differs significantly between preterm and term-born infants up to the age of 4.5 years, with preterm infants experiencing lower BHI. However, beyond this age, no significant differences were observed until mid-adulthood, suggesting a stable and sustained catch-up in bone health among preterm infants. Notably, no association between BHI and fracture status was identified in this study.

By utilizing an extended longitudinal design spanning several decades, we provide novel evidence that moderate to late preterm infants experience a catch-up in bone health during early childhood, which is maintained into mid-adulthood. Given that approximately 85% of preterm children fall into this category^1^, this finding is of particular importance. First, it provides reassurance to parents that the bone health of their former moderate to late preterm child will likely, over time, align with that of term-born peers. Second, it carries potential clinical implications, particularly for children who do not achieve catch-up in BHI by early childhood, allowing for early identification and targeted interventions. Our results align with previous studies focusing on similar moderate to late preterm cohorts. For instance, Dalziel et al. and Breukhoven et al. reported no significant differences in bone health between preterm and term-born individuals in the age range of 18–31 years using a cross-sectional design^20,21^. Additionally, a longitudinal study by Zhao et al. observed accelerated growth in bone health in the first 12 months, suggesting that the catch-up process begins early but may not be fully complete within this timeframe^26^. The present study extends these findings by providing a more comprehensive, long-term perspective on bone health development.

In contrast, studies focusing on very low birth weight (VLBW) or extremely preterm infants have reported different results. Several studies reported deficits in bone health in early adulthood (ages 18 to 35 years), suggesting the absence of catch-up in bone health in these high-risk cohorts^15–19^. Most of these studies have a cross-sectional design, limiting their ability to capture the dynamic, complex process in bone health. This underscores the need for longitudinal, comprehensive studies specifically targeting this high-risk group, as our findings are not directly generalizable to this population.

In our cohort, no significant association between BHI and fracture status was detected based on self-reported fracture data available for approximately half of the participants in mid-adulthood. This contrasts with findings in the Generation R study, which demonstrated an 11% increased risk of prevalent fractures per one standard deviation decrease in BHI^43^. Fracture risk between preterm and term-born individuals has generally not been shown to differ during toddlerhood, childhood or early adulthood^44–46^. Interestingly, a follow-up study up to the age of 29 years found that individuals born extremely preterm had an even lower fracture risk^45^, suggesting that factors beyond bone health contribute significantly to fracture susceptibility. Potential explanations include more cautious behavior, increased parental protection and lower participation in physical activity among preterm-born children^45^. Conversely, Michaud et al. reported preterm birth to be associated with higher risk of fracture-related hospitalizations within the first 18 months^47^. It is important to investigate whether fracture risk, once again, becomes a concern in preterm individuals as they enter old age and osteoporotic fractures in particular become more common^48^.

In summary, the current findings highlight the importance of monitoring bone health, particularly in preterm-born individuals who do not achieve catch-up in bone health early in life. Consequently, a lifespan perspective on preterm birth is needed.

A key strength of this study is its unique longitudinal design over several decades, providing a rare and valuable dataset that offers insights into the dynamics of bone health from infancy to mid-adulthood using advanced statistical modeling^38,40^. To our knowledge, this is the most comprehensive dataset available and the only study examining bone health over such an extended period. Considering the fact that growth patterns may influence BMD in preterm children^49^, using BHI as a growth-independent measure of bone health is another strength of this study.

This study also has limitations worth mentioning. The assessment of bone health in children remains a subject of ongoing debate^50^. BHI has been shown to correlate with Dual-energy X-ray Absorptiometry (DXA) and peripheral Quantitative Computed Tomography (pQCT)^31,43,50–52^ and can be applied across various clinical settings and populations^53–58^. However, it is not considered the gold standard for bone health assessment. Given its low radiation exposure, affordability, and practicality, BHI represents a promising option for longitudinal assessments starting in early childhood to assess bone health^50^, especially in at-risk populations, such as preterm-born infants. Further research is needed to clarify its clinical utility and establish its role in routine practice.

Several studies have investigated the role of nutrition and early feeding type on bone health of former preterm infants in different populations^24,25,59^. Because no details on early feeding types were available in our cohort, we could not investigate this. In addition, data on pubertal timing or stage were not available, which limits our ability to directly account for potential effects of puberty on bone development. Previous studies have suggested that both small for gestational age and early postnatal weight gain may be associated with earlier pubertal onset, indicating that differences in maturational timing could influence later bone outcomes^60,61^. However, in our cohort, analyses based on bone age instead of chronological age yielded comparable results, suggesting that maturational differences are unlikely to have substantially influenced our findings.

As the digitization of the ZLS dataset is still ongoing^27^, additional information (e.g., on early feeding type) may become available in the future, making further investigations into these important questions feasible.

In our cohort, gestational age was determined based on the last menstrual period, which was self-reported and may therefore include some degree of inaccuracy.

Our study was not designed to treat fracture history as a potential causal predictor of childhood BHI. However, examining whether BHI dynamics differ between those who eventually sustain a fracture and those who do not remains informative and was therefore included into this descriptive study. Fractures were self-reported and not verified by radiographic images, potentially introducing recall bias and misclassification. Additionally, missing fracture data for approximately half of the participants in mid-adulthood reduced the sample size and may have limited statistical power. Similarly, subgroup analyses focusing on high-risk children, such as those with necrotizing enterocolitis or juvenile idiopathic arthritis^53,62^, were not feasible due to small sample sizes and missing data, further limiting the detection of potential associations.

In conclusion, moderate to late preterm-born children demonstrate the capacity for a sustained catch-up in bone health in early childhood that persists into mid-adulthood. Nevertheless, it remains unclear how early-life differences affect bone health in advanced age and whether this catch-up persists or whether preterm-born individuals experience an accelerated decline in bone health later in life. Future research should prioritize longitudinal investigations extending into older age groups to expand these findings, while also examining extremely preterm-born individuals separately, as they may exhibit distinct long-term risks. Additionally, targeted interventions for preterm-born children who do not achieve early catch-up in bone health should be explored to reduce potential long-term health risks.

## Supplementary Information

Below is the link to the electronic supplementary material.

Supplementary Material 1

## Data Availability

The datasets generated and/or analysed during the current study are not publicly available due to the approval granted by the ethical committee of the Canton of Zurich, Switzerland for the Zurich Longitudinal Studies (Basec No. 2018–00,686) not allowing the publication of the raw data online but are available from the corresponding author on reasonable request.
