# Supplementary material for: Longitudinal course of bone health from infancy to mid-adulthood in preterm and term-born individuals
Source: Sci Rep. 2025 Nov 26;15:45371. doi: 10.1038/s41598-025-29584-0 (PMC12749154; doi:10.1038/s41598-025-29584-0)
Supplement: Supplementary file 1 — Supplementary Material 1 [file 41598_2025_29584_MOESM1_ESM.docx]

**Supplemental Material**

*Longitudinal Course of Bone Health from Infancy to Mid-Adulthood in Preterm and Term-Born Individuals*

*Brecher, Julie, MMed^1^; Chaouch, Aziz, MSc^2^; Konrad, Daniel, PhD^3,4,5^; Eichelberger, Dominique A., PhD^1^, Latal, Beatrice, MD MPH^1,3,4^, Jenni, Oskar G., MD^1,3,4^; Lenherr, Nina, MD^3,5^* & Wehrle Flavia M., PhD^1,3,4^**

***Supplementary Figure 1:*** *Distribution of number of taken hand X-rays.*


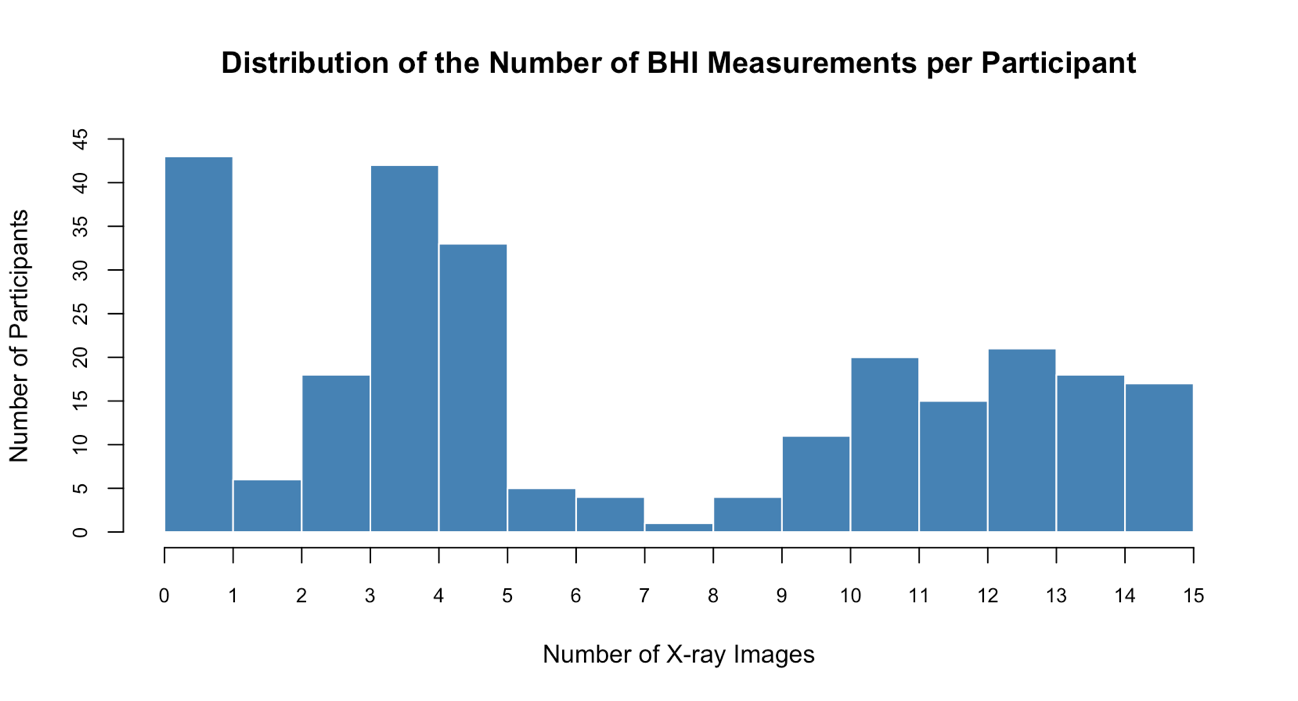


***Supplementary Figure 2:*** *shows the estimated difference of the predicted BHI values over bone age based on the GEE model (BHI~preterm*ns(bone age, df=5 + sex). The solid line represents the estimated difference, while the shaded area corresponds to the 95% confidence interval. Values above the dashed line indicate higher BHI values for preterm individuals. Areas where the confidence interval does not include the zero line are statistically significant.*

*For following analysis, 1474 radiographs from 208 participants were included, comprising 116 preterm and 92 term-born individuals, as for those bone age values were available.*

*The best-fitting GEE model used natural cubic splines with 5 degrees of freedom (QICu=262.3). The model revealed a significant main effect of preterm birth on BHI, with preterm individuals exhibiting lower BHI compared to term-born individuals (β=-0.401, SE=0.134, p=0.0028). Bone age had a significant impact on BHI, with nonlinear associations indicating both positive and negative changes depending on the bone age segment. Rather than interpreting individual spline coefficients, the overall trend suggests that BHI increases at later bone ages, while no significant changes were observed at earlier bone ages. Sex was not significantly associated with BHI (β=-0.011, SE=0.043, p=0.80). Significant interactions between preterm status and bone age indicate that preterm individuals follow a distinct age-related trajectory in BHI. As illustrated in the figure, the GEE model simulation showed a significantly lower BHI in preterm infants up to a bone age of 2.75 years. Beyond this bone age, no significant differences in BHI between preterm and term-born infants were detected. Based on the estimated difference, preterm infants achieve catch-up in BHI by approximately 4.5 years. Excluding one individual with potential osteoporotic fractures did not alter the findings (data not shown).*

***Supplementary Figure 3:*** *Estimated difference of the predicted BHI values over age based on the GEE model (BHI~preterm*ns(age, df=5) + fracture status*ns(age, df=5) + sex). The solid line represents the estimated difference, while the shaded area corresponds to the 95% confidence interval. Values above the dashed line indicate higher BHI values for individuals with no sustained fracture. Areas where the confidence interval does not include the zero line are statistically significant.*
